# Primary Thoracic Epidural Melanoma : A Case Report

**DOI:** 10.4184/asj.2010.4.1.48

**Published:** 2010-04-26

**Authors:** Kwang-Wook Jo, Seong-Rim Kim, Sang-Don Kim, Ik-Seong Park

**Affiliations:** Department of Neurosurgery, Bucheon St. Mary's Hospital, The Catholic University of Korea College of Medicine, Bucheon, Korea.

**Keywords:** Central nervous system, Primary spinal melanoma, Thoracic lesion

## Abstract

A 68-year-old woman with progressive paraparesis and altered sensation lasting approximately five days was admitted to our clinic. Magnetic resonance imaging (MRI) revealed an advanced stage T7-8 epidural mass ventral to the spinal cord, which was believed to be a metastatic tumor considering the patient's age. A highly enhanced epidural mass and pedicle appeared during the MR scan. However, the pathologic findings were compatible with the diagnosis of a primary meningeal melanocytic tumor. Primary epidural melanomas are extremely rare lesions. This case was finally diagnosed as a primary thoracic spinal epidural melanoma.

## Introduction

Most malignant melanomas in the central nervous system (CNS) are metastatic lesions [1]. Most spinal melanomas are intradural with or without extradural components. Primary extradural spinal melanomas are extremely rare lesions [1,2]. To date, only 5 cases of primary extradural spinal melanomas have been reported [3]. Among these, only one occurred in the spinal epidural space [3]. We report a case of primary thoracic spinal extradural melanoma.

## Case Report

**Fig. 1:**
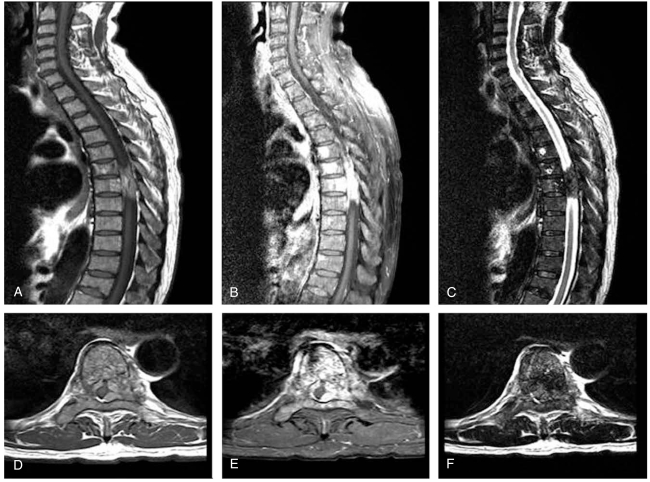
T1-weighted magnetic resonance imaging demonstrating a T7-8 epidural mass ventral to the spinal cord (**A**: sagittal, **D**: axial) with high enhancement (**B**: sagittal, **E**: axial); the lesion showed low signal intensity on the T2-weighted images (**C**: sagittal, **F**: axial).

A 68-year-old woman with progressive paraparesis and altered sensation lasting approximately five days was admitted to our clinic. Neurologically, the patient showed paraparesis (IV/III), hypesthesia and hypoalgesia below the T9 dermatome, as well as preserved proprioception. The knee-jerk was hyper-reflexive and ankle clonus was identified bilaterally. T1-weighted magnetic resonance imaging (MRI) demonstrated an advanced T7-8 epidural mass ventral to the spinal cord. The T2-weighted images revealed low signal intensity for the lesion (Fig. 1).

**Fig. 2:**
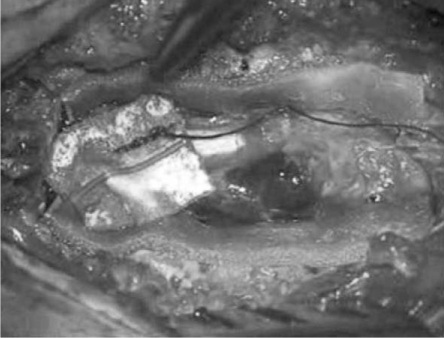
As soon as laminectomy had been performed, an epidural, firm, a black neoplasm was encountered, which invaded the pedicle and distorted the spinal cord due to ventral involvement.

**Fig. 3:**
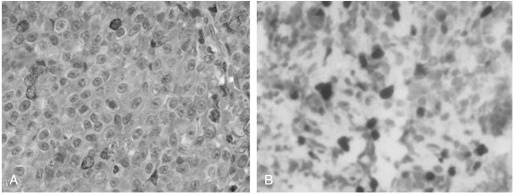
A pathologic examination revealed small round blue spindle tumor cells with melanin pigmentation and occasional epitheloid melanocytes with a predominance of macronuclei: necrosis, hemorrhage, and nuclear. Cellular pleomorphism were absent, the number of mitotic figures were low **(A)**, and the Ki-67 index was measured 8% **(B)**.

The mass was believed to be a metastatic tumor based on the patient's age and MRI findings (epidural, highly enhanced, and pedicle involvement). Chest and abdominal CT, gastroscopy, colonoscopy, bone scan, tumor marker levels, and mammography were used to find the primary tumor focus but were unsuccessful. She underwent surgery via the posterior, transpedicular approach. After the laminectomy, a firm, black, epidural neoplasm was encountered, which had invaded the left pedicle, encasing and distorting the spinal cord (Fig. 2). The mass was removed enough to decompress the spinal cord. The mass was confirmed to be an extradural mass in the surgical field. A pathologic examination revealed small round blue spindle tumor cells with melanin pigmentation and some epitheloid melanocytes with a predominance of macronuclei. Necrosis, hemorrhage, nuclear and cellular pleomorphisms were absent, and the number of mitotic figures were low. The Ki-67 index was 8% (Fig. 3). These findings were compatible with those of a primary malignant melanoma. After the procedure, she showed improved neurological conditions. The neurological examination showed that her diffuse lower extremity weakness had improved IV+ and her T9 sensory level for pain and temperature sensation was normalized. No primary malignant melanoma was observed through dermatologic and ophthalmologic examinations. On the 4th day of surgery, the patient presented with hypesthesias below the T9 sensory dermatome. The follow-up MRI revealed fluid collection at the surgical site. Reoperation and radiosurgery was recommended but she refused. The patient was transferred to the oncology department for chemotherapy and radiotherapy. Sixteen days after surgery, the patient underwent palliative spinal radiotherapy 30 cGY/10 fraction at T5-T9 and L2-S2. Twenty days after radiotherapy, she underwent chemotherapy using two cycles of interferon. Her responses to radiotherapy and chemotherapy were not good, and she expired due to pneumonia 6 months after chemotherapy and radiotherapy.

## Discussion

Melanoma is a malignant neoplasm arising from melanocytes. Melanocytes are melanin-producing cells that arise from the neural crest during embryogenesis and migrate to the skin, mucous membranes, and CNS [1]. In the CNS, melanocytes are most numerous in the leptomeninges at the anterior and lateral surfaces of the spinal cord, brain stem, and base of the brain [3], This case showed that melanomas found only in the epidural space encased the spinal cord, which suggests that melanoma might arise in the anterior superficial surfaces of the meninges. The MRI findings of melanomas vary considerably depending upon the number of melanin-containing cells and the presence or absence of hemorrhage [4,5]. In a typical melanotic melanoma, melanin has a paramagnetic effect derived from the presence of stable organic radicals inside. The unpaired electrons of these free radicals interact with the water protons, resulting in a shortening of both T1 and T2 relaxation times, producing hyperintensity on the T1-weighted images and hypointensity on the T2-weighted images. In our case, the MRI findings were similar to those normally observed for melanoma. However, the MRI findings in our case were compatible with a metastatic tumor. Therefore, we could not rule out a metastatic tumor due to old age, epidural lesion, tumor maturity, or pedicle involvement. Unfortunately, these findings are found in less than 50% of typical melanomas [4,5]. According to the Hayward classification, a diagnosis of primary melanoma is based on the absence of a melanoma outside the CNS, an absence of a melanoma in other CNS sites, and histological confirmation of a melanoma [6]. This case was diagnosed as a primary melanoma based on these criteria.

Meningiomas, schwannomas, and angiomatous lesions, such as cavernous hemangiomas or hemangiopericytomas, are rarely purely extradural and may show intratumoral hemorrhaging [3,7]. Meningiomas and schwannomas are well-demarcated lesions that may also contain melanin pigment and reveal hyperintensity on the T1 images [3,8], Although most spinal meningiomas are intradural, King et al. [7] reported that approximately 2.5% of these are purely extradural. Another melanin-containing lesion, meningeal melanocytoma, is rarely found alone, and may be indistinguishable from a melanoma, even though it is found more commonly in the cervical region [9].

For primary CNS melanocytic neoplasms, a complete tumor resection is preferred because it can cure well-differentiated and intermediate-grade melanocytomas (IMGs) and most melanomas [10,11]. Radiotherapy is recommended for the incomplete resectioning of IMGs and melanomas. The risk of recurrence of low-grade melanocytomas is less clear and careful observation may be needed for the same situation because recurrent tumors may require surgical treatment prior to radiation [10,11]. In our case, a complete tumor resection was believed to be dangerous because the mass was located in the ventral portion of the thoracic spinal cord. The mass was removed partially just enough to decompress the spinal cord. Chemotherapy and radiotherapy were performed to treat the remainder of the tumor.

Primary CNS melanomas are relatively uncommon lesions compared to metastatic melanomas in the CNS. Once metastases, including drop metastases from pigmented medulloblastomas, have been excluded, the differential diagnosis includes pigmented meningiomas and schwannomas (solitary or as part of the Carney complex), as well as other pigmented CNS tumors, such as ependymoma and pineoblastoma and systemic diseases, such as lymphomas [10-12].

## References

[1] Kwon SC, Rhim SC, Lee DH, Roh SW, Kang SK (2004). Primary malignant melanoma of the cervical spinal nerve root. Yonsei Med J.

[2] Naing A, Messina JL, Vrionis FR, Daud AI (2004). Uncommon manifestations of common malignancies: case 3. Malignant melanoma arising from a spinal nerve root. J Clin Oncol.

[3] Unal B, Castillo M (2007). MRI features of a primary thoracic epidural melanoma: a case report. Clin Imaging.

[4] Isiklar I, Leeds NE, Fuller GN, Kumar AJ (1995). Intracranial metastatic melanoma: correlation between MR imaging characteristics and melanin content. AJR Am J Roentgenol.

[5] Woodruff WW, Djang WT, McLendon RE, Heinz ER, Voorhees DR (1987). Intracerebral malignant melanoma: high-field-strength MR imaging. Radiology.

[6] Hayward RD (1976). Malignant melanoma and the central nervous system: a guide for classification based on the clinical findings. J Neurol Neurosurg Psychiatry.

[7] King AT, Sharr MM, Gullan RW, Bartlett JR (1998). Spinal meningiomas: a 20-year review. Br J Neurosurg.

[8] Culhaci N, Dikicioglu E, Meteoglu I, Boylu S (2003). Multiple melanotic schwannoma. Ann Diagn Pathol.

[9] Alameda F, Lloreta J, Galito E, Roquer J, Serrano S (1998). Meningeal melanocytoma: a case report and literature review. Ultrastruct Pathol.

[10] Brat DJ, Giannini C, Scheithauer BW, Burger PC (1999). Primary melanocytic neoplasms of the central nervous systems. Am J Surg Pathol.

[11] Rades D, Heidenreich F, Tatagiba M, Brandis A, Karstens JH (2001). Therapeutic options for meningeal melanocytoma: case report. J Neurosurg.

[12] Johnson MD, Powell SZ, Boyer PJ, Weil RJ, Moots PL (2002). Dural lesions mimicking meningiomas. Hum Pathol.

